# Formulation-Dependent Physical, Mechanical, and Water-Exposure Behavior of Hemp-Based Mycelium Composites with Polymeric Additives

**DOI:** 10.3390/ma19204277

**Published:** 2026-10-10

**Authors:** Ewa Borucinska-Parfieniuk, Małgorzata Kowalska, Madhavi H. Dalsaniya, Adrian Dubicki, Magdalena Pantol, Wojciech Grodzki, Małgorzata Krasowska, Jolanta Piekut, Eugene Yurchenko, Ewa Zapora, Izabela B. Zglobicka

**Affiliations:** 1 Faculty of Mechanical Engineering, Bialystok University of Technology, Wiejska 45B, 15-351 Bialystok, Poland; ewa.borucinska-parfieniuk@sd.pb.edu.pl (E.B.-P.); madhavi.dalsaniya@pb.edu.pl (M.H.D.); a.dubicki@pb.edu.pl (A.D.); magdalena.pantol@sd.pb.edu.pl (M.P.); w.grodzki@pb.edu.pl (W.G.); 2 Faculty of Civil Engineering and Environmental Sciences, Bialystok University of Technology, Wiejska 45E, 15-351 Bialystok, Polandm.krasowska@pb.edu.pl (M.K.); j.piekut@pb.edu.pl (J.P.); yauheni.yurchanka@pb.edu.pl (E.Y.); e.zapora@pb.edu.pl (E.Z.)

**Keywords:** biofabricated materials, fungal biocomposites, hemp hurds, hot pressing, polymer reinforcement, moisture-induced swelling, dimensional stability, specimen-scale heterogeneity

## Abstract

**Highlights:**

- Apparent density showed a positive specimen-level association with maximum compressive stress.
- Similar mean densities were associated with different mechanical responses.
- Water uptake, dimensional swelling, and wet-state integrity followed different trends.
- Pronounced specimen-to-specimen variability reflected structural heterogeneity.
- Mycelium composite design requires balancing mechanical performance and water-exposure stability.

**Abstract:**

Hemp-based mycelium composites are promising bio-based materials, but their heterogeneous structure may strongly affect mechanical performance and stability under water exposure. Six hot-pressed composites based on hemp hurds and chopped hemp straw, colonized with *Pleurotus ostreatus* or *Ganoderma applanatum*, were investigated. Depending on formulation, spent coffee grounds, thermoplastic or plasticized starch, and polymeric components introduced as granules, film flakes, or poly(ε-caprolactone) meshes were incorporated. Apparent density, water absorption, dimensional changes during immersion, compression, and three-point bending were evaluated. Mean apparent density ranged from 0.69 to 0.96 g × cm^−3^ and showed a positive specimen-level association with maximum compressive stress within the investigated plates (Spearman’s ρ = 0.845, *n* = 30). Because only one plate was manufactured per formulation, the observed formulation-specific property profiles represent exploratory observations within the investigated manufactured plates rather than population-level estimates of formulation effects or manufacturing reproducibility. The highest mean maximum compressive stress was 5.7 ± 1.2 MPa, whereas the highest mean maximum flexural stress and flexural modulus were 4.6 ± 2.1 MPa and 627 ± 285 MPa, respectively. Water uptake, dimensional swelling, and retention of measurable integrity followed different formulation-dependent trends; the greatest through-thickness swelling did not necessarily correspond to the fastest loss of cohesion. Overall, density, mechanical response, water uptake, dimensional deformation, and wet-state integrity were related but distinct descriptors of material performance, highlighting the need for multi-property optimization.

## 1. Introduction

Mycelium-based composites are biofabricated, typically lightweight and porous materials formed when fungal hyphae colonize lignocellulosic substrates and bind their particles or fibers into a cohesive structure [1,2]. The lignocellulosic substrate serves both as a nutrient source for fungal growth and as the structural framework of the resulting composite. Because such substrates may originate from agricultural and forestry residues, mycelium composites are of interest for waste valorization and circular-material strategies [1,2]. Their low density and useful thermal and acoustic properties have stimulated interest in applications such as protective packaging, insulation, lightweight panels, and other non-structural products [1,2,3]. However, their environmental performance depends not only on biological origin but also on raw-material sourcing, processing conditions, service life, and end-of-life scenario [4,5,6]. Mycelium composites should therefore be regarded as a configurable family of bio-based engineering materials whose properties and environmental performance depend strongly on composition, processing, and intended application.

The lignocellulosic substrate determines both the biological and structural development of mycelium composites. Its chemical composition, particle size, fiber length, packing, and accessibility to fungal colonization influence pore structure, local consolidation, and the extent of biological binding [7,8,9]. Hemp hurds and chopped hemp straw are particularly attractive feedstocks because they are lightweight agricultural residues that can be processed into particles and fibers of different dimensions [7,10]. These structural features affect density and mechanical response, while the cellulose- and hemicellulose-rich lignocellulosic phase also promotes interaction with water. Moisture sensitivity is therefore governed by both substrate chemistry and the geometry and connectivity of the internal pore network [7,8,11,12]. Hemp-derived constituents should consequently be regarded as an active structure-forming phase rather than merely as inert filler.

During incubation, fungal hyphae colonize accessible substrate surfaces, extend through the pore space, and bridge neighboring lignocellulosic particles and fibers, thereby forming a three-dimensional network that provides cohesion to the composite [1,13,14,15]. The effectiveness of this biological binding depends on the extent and spatial uniformity of colonization, continuity of the hyphal network, and quality of contact between the mycelium and substrate. Local differences in colonization, substrate packing, moisture distribution, nutrient availability, and incubation conditions may therefore generate regions with different degrees of consolidation and mechanical response [2,8,16,17]. Spatial heterogeneity should consequently be considered an inherent feature when relating processing conditions to the physical and mechanical behavior of mycelium composites.

Moisture sensitivity and limited reproducibility remain major challenges for the broader engineering use of mycelium-based composites. Their hydrophilic lignocellulosic constituents and interconnected pore networks promote moisture sorption and liquid-water uptake, which may lead to swelling, dimensional instability, deterioration of mechanical properties, and loss of material cohesion [8,18,19,20]. Although a dense aerial mycelial layer may increase surface hydrophobicity, this effect does not necessarily reflect the response of the entire porous composite during prolonged water exposure [8,21]. At the same time, local differences in substrate packing, moisture distribution, compaction, and fungal colonization may contribute to substantial variability among nominally identical specimens [2,8,17,20]. Improving moisture tolerance while reducing specimen-to-specimen variability therefore remains a key challenge in the development of mycelium-based engineering materials.

Several strategies have been explored to modify the performance of mycelium-based composites, including changes in substrate composition and particle geometry, incorporation of food-waste and starch-based components, densification, thermal post-processing, and the introduction of discrete or continuous reinforcing phases [9,10,22,23,24,25,26]. Spent coffee grounds represent an abundant food-waste stream that may influence substrate packing, density, fungal growth, and mechanical or water-related behavior [27]. Starch-based components can also perform different functions depending on their physical form. Native or plasticized starch may primarily act as a substrate component, whereas thermoplastic starch can additionally function as a distinct polymeric phase [22,28]. Polymeric reinforcement introduces an additional structural variable, as its effect may depend not only on chemical composition but also on continuity, geometry, distribution, orientation, and position within the mycelium-bound structure [25,26,29].

Although previous studies have addressed lignocellulosic substrate selection, fungal growth, starch-containing formulations, food-waste-derived components, and different reinforcement strategies [9,10,22,23,24], comparatively little attention has been given to the combined physical, mechanical, and water-exposure behavior of mycelium composites containing polymeric components in different physical forms and spatial arrangements. Dispersed polymeric phases such as granules or film flakes and localized continuous meshes may interact differently with the heterogeneous lignocellulosic–mycelial structure and may therefore produce different combinations of stiffness, strength, dimensional response, and structural integrity [25,26,29].

The working hypothesis was that the investigated material systems would exhibit distinct combinations of mechanical and water-exposure responses and that apparent density alone would not fully account for their overall performance.

The present study comparatively evaluates six hemp-based mycelium composite material systems containing different combinations of spent coffee grounds, starch, and poly(ε-caprolactone) components. The materials were characterized in terms of apparent density, water absorption and dimensional response during prolonged immersion, compressive behavior, and three-point flexural properties. Particular attention was given to specimen-to-specimen variability and to the relationships among density, mechanical performance, water uptake, dimensional swelling, and retention of measurable structural integrity.

## 2. Materials and Methods

### 2.1. Raw Materials and Fungal Species

Strains of two fungal species, *Pleurotus ostreatus* FCBUT 2-9 and *Ganoderma applanatum* FCBUT 2-18, were used for the production of the mycelium composites. Both strains originated from the fungal culture collection of the Institute of Forest Sciences, Białystok University of Technology, Poland.

The lignocellulosic fraction consisted of hemp hurds and hemp straw obtained from agricultural and agro-food processing residues generated locally in the Podlaskie Voivodeship, Poland. Prior to composite preparation, the hemp straw was mechanically chopped using an RD-150 shredder (TechnoMaszBud, Słupno, Poland). The resulting straw fraction consisted predominantly of particles approximately 4–15 mm in length and 0.5–4 mm in width and additionally contained a fibrous fraction approximately 10–20 mm in length and 0.01–0.02 mm in diameter. Hemp hurds consisted predominantly of particles approximately 1–4 mm in length and 0.5–1.5 mm in width.

Depending on the formulation, the lignocellulosic substrate was supplemented with spent coffee grounds and starch- or polymer-based components. Poly(ε-caprolactone) (PCL), thermoplastic starch (TPS), plasticized potato starch, and a biodegradable thermoplastic film-grade material were used as polymeric or starch-based components.

PCL (CAS 24980-41-4, Sigma-Aldrich, St. Louis, Missouri, USA) had a melt flow index (MFI) of 2–4 g × 10 min^−1^ (160 °C, 5 kg). Thermoplastic starch was used in the form of the commercial compostable extrusion-grade material envifill^®^ TPS (Grupa Azoty S.A., Tarnów, Poland), based on potato starch and supplied in granular form. The material had an MFI of 4–8 g × 10 min^−1^ (160 °C, 5 kg).

Plasticized starch (Grupa Azoty S.A., Tarnów, Poland) was prepared from potato starch, glycerol, and water at proportions of 70, 20, and 10 wt.%, respectively. The components were mixed at 40 °C for approximately 30 min using a PRS planetary mixer (ERWEKA GmbH, Langen, Germany). The resulting mixture was transferred to a sealed container and conditioned for 24 h prior to use.

The polymer film flakes used in formulations 2 and 3 were prepared from film produced from commercial envifill^®^ F40 granulate (Grupa Azoty S.A., Tarnów, Poland). The detailed chemical composition of the commercial envifill^®^ F40 grade was not disclosed by the supplier and was therefore treated in the present study as a proprietary biodegradable thermoplastic formulation. A film with a nominal thickness of 35 µm was manufactured by blown-film extrusion using a MICROEX BLOWN laboratory extrusion line (EUR.EX.MA S.r.l., Tradate, Italy). The resulting film was subsequently fragmented into flakes using a custom-built laboratory twin-shaft shredder (Grupa Azoty “Automatyka”, Tarnów, Polska).

In the present study, TPS and plasticized starch were treated as distinct material components: TPS was a commercial thermoplastic material supplied in granular form, whereas plasticized starch was prepared separately and used in powder form.

PCL was selected as a biodegradable thermoplastic combining suitable melt processability with the possibility of fabrication by material-extrusion 3D printing. Its processing characteristics also enabled the polymeric component to be introduced in different initial physical configurations, including dispersed granules and continuous mesh structures, thereby providing an additional structural variable within the investigated bio-based composite systems.

### 2.2. Composite Design and Formulation Strategy

Six mycelium composite formulations were investigated (Table 1). Formulation 5 was also produced within the original experimental series; however, the resulting material exhibited insufficient macroscopic cohesion and disintegrated during handling, preventing reproducible specimen preparation. It was therefore excluded from subsequent quantitative characterization. The formulations differed in fungal species, the presence of spent coffee grounds, the type of starch-based component, and the physical form and spatial arrangement of the polymeric phase. The polymeric phase was introduced as poly(ε-caprolactone) (PCL) meshes, dispersed PCL granules, or dispersed polymer film flakes. The quantitative composition of the investigated formulations per mold is provided in Table 2.

Because several compositional and structural variables differed simultaneously among the formulations, the materials were treated as complete material systems rather than as independent levels of individual constituents. Accordingly, the study was designed as a comparative assessment of formulation-specific property profiles rather than as a factorial evaluation of the effects of individual components.

One composite plate was produced for each successfully consolidated formulation. Each plate originated from a single mold and cultivation batch. All specimens assigned to a given formulation were subsequently cut from the corresponding single plate and therefore represent within-plate subsamples rather than independent manufacturing replicates.

### 2.3. Preparation of PCL Meshes

PCL meshes were fabricated from neat PCL by material-extrusion 3D printing using a Bambu Lab P1S printer (Shenzhen Tuozhu Technology Co., Ltd., Shenzhen, China). PCL was processed at a nozzle temperature of 180 °C using a 0.4 mm nozzle. The build-plate temperature was set to 30 °C, and a poly(vinyl alcohol) (PVA)-based adhesive was applied to the build plate to improve first-layer adhesion. The layer height was 0.2 mm, and the printing speed was 150 mm *×* s^−1^. The fabricated meshes had a regular structure with a nominal opening size of 1 × 1 mm, an overall thickness of 2 mm, and external dimensions of 200 × 200 mm. The mass of the individual PCL meshes was not recorded during fabrication. The meshes were subsequently incorporated as continuous polymeric elements positioned at selected outer surfaces of the composites according to the configuration specified for each formulation in Table 1.

### 2.4. Substrate Preparation, Sterilization, and Inoculation

The components of each formulation were thoroughly mixed to obtain a macroscopically homogeneous substrate. Water was then added to obtain a moisture level suitable for molding and fungal growth. The amount of added water depended on substrate composition and ranged from 1.85 L × kg^−1^ of dry material for the denser substrate, containing a higher proportion of spent coffee grounds and hemp hurds, to 3.3 L × kg^−1^ for the less densely packed substrate, containing a higher proportion of hemp straw. Formulation-specific water additions were not recorded separately and therefore could not be reliably reconstructed retrospectively. The prepared mixtures were transferred directly into thin-walled metal molds measuring 23 × 23 × 6 cm, which served both as shaping elements and cultivation containers. The mold bottoms were perforated with 2.5 mm diameter holes spaced 2.5 cm apart.

The filled molds were covered with aluminium foil and sterilized in an autoclave (Advantage-Lab AL02-12-100, Schilde, Belgium) at 121 °C for 30 min. After cooling to room temperature, the substrates were inoculated under aseptic conditions in a laminar-flow cabinet using the fungal strain assigned to the respective formulation.

The inoculum (spawn) was added at 200 mL per mold, corresponding to approximately 1:16 (*v*/*v*). Before inoculation, the spawn was broken into approximately 1.5–3 cm pieces and distributed throughout the substrate to promote uniform colonization. The spawn had previously been prepared in 1 L glass jars containing a sterilized substrate composed of hemp hurds supplemented with ground barley malt (10 g × kg^−1^) and water (3.3 L × kg^−1^). The spawn substrate was sterilized at 121 °C for 30 min, inoculated with an agar culture of the respective fungus, and incubated for 3–4 weeks at 18 °C before use.

### 2.5. In-Mold Growth and Composite Formation

Following inoculation, the materials were incubated in the molds for 14 days in a KK 400 SMART PRO climatic chamber (Pol-Eco^®^ sp.k., Wodzisław Śląski, Poland) at 18 °C and 70% relative humidity. During this period, fungal colonization progressed throughout the lignocellulosic substrate, binding the substrate components and producing a cohesive mycelium-based structure.

Composite shaping occurred concurrently with fungal growth because the substrates remained confined within the molds throughout the cultivation period. Depending on the formulation, polymeric components were either dispersed within the substrate or positioned as PCL meshes at selected outer surfaces, as specified in Table 1. After 14 days of cultivation, the formed mycelium composite plates were removed from the molds and subjected to post-growth drying and hot pressing, as described in Section 2.6.

### 2.6. Post-Growth Processing and Specimen Preparation

Following the cultivation period, the mycelium-based composites were dried in a BINDER drying oven (BINDER, Tuttlingen, Germany) at 40 °C for 24 h to reduce excess moisture. The dried materials were subsequently hot-pressed using a LaBECON 300 laboratory press (Fontijne Presses, Rotterdam, The Netherlands).

**Figure 1:**
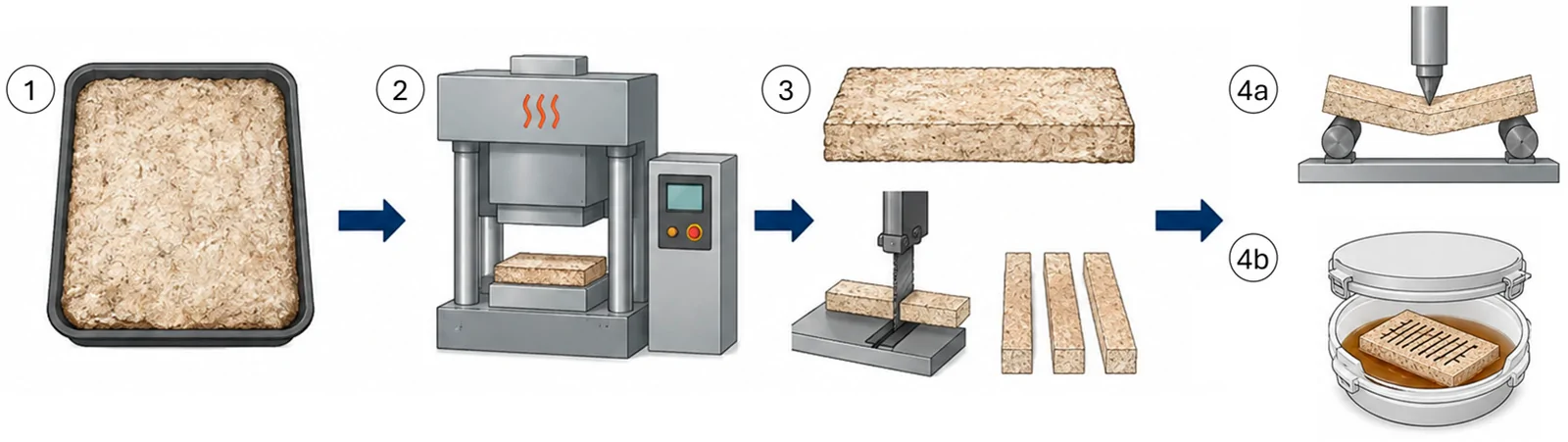
Schematic overview of post-growth processing and specimen preparation: (**1**) mycelium-bound plate after cultivation; (**2**) hot pressing; (**3**) cutting of test specimens; and (**4**) selected testing routes: (**a**) three-point bending and (**b**) water-immersion testing.

The overall post-growth processing procedure is schematically presented in Figure 1.

Hot pressing was performed at a platen temperature of 100 °C for 2 h. The final platen separation was set to 5 mm as the nominal consolidation setting. This value represented the final separation between the press platens during hot pressing and should not be interpreted as an imposed exact final specimen thickness. The actual consolidated-plate thickness showed local variation associated with the heterogeneous structure and surface morphology of the material. Consolidation was controlled by the final platen separation rather than by a prescribed pressure or force. Applied force and pressure were neither independently controlled nor recorded during the pressing cycle. At the end of the hot-pressing cycle, the plates were removed from the press without an additional cooling step in the press.

Following hot pressing, the consolidated plates intended for mechanical testing were stored in a laboratory drying oven at 37 °C for approximately 24 h before specimen preparation. Compression and three-point bending specimens were tested immediately after cutting, without an additional conditioning step at controlled relative humidity.

For consistent identification of plate orientation, the surface facing the covering foil was designated as the upper surface, whereas the surface facing the metal tray was designated as the lower surface. Cross-sectional views corresponded to the through-thickness direction of the plates. Representative macroscopic images of the consolidated plates were recorded prior to specimen cutting and are provided in Supplementary Materials, Figure S1.

The consolidated plates were subsequently cut into specimens for apparent density, water-absorption, compression, and three-point bending measurements. Specimen geometry and testing conditions are described separately for each characterization method in Section 2.7.

### 2.7. Physical and Mechanical Characterization

#### 2.7.1. Apparent Density

The apparent density of the mycelium composites was determined using the geometric method. Five specimens were analyzed for each material formulation *(n* = 5).

The mass of each specimen was measured three times using a RADWAG AS 220.R2 analytical balance (RADWAG, Radom, Poland). Specimen length, width, and thickness were measured three times using a Mitutoyo Absolute AOS Digimatic CD-15DAX digital caliper (Mitutoyo Corporation, Kanagawa, Japan). Owing to the irregular and locally uneven surfaces of the mycelium composites, dimensional measurements were taken at representative locations on each specimen.

For each specimen, the mean mass and mean length, width, and thickness were calculated from the repeated measurements. The apparent density of an individual specimen, ρ*_app_*_,*i*_, [g × cm^−3^] was calculated according to Equation (1):

(1) $$\rho_{\mathrm{app},i}=\frac{\bar{m_{i}}}{l_{i}w_{i}t_{i}}$$

where *m_i_* is the mean mass of specimen *i* [g], and *l_i_*, *w_i_*, *t_i_* are mean length, width and thickness, respectively [cm].

The mean apparent density of each material formulation, ${\bar{\rho }}_{\mathrm{app}}$, was subsequently calculated from the apparent densities of the individual specimens according to Equation (2):

(2) $$\bar{\rho_{\mathrm{app}}}=\frac{1}{n}\sum_{i=1}^{n}\rho_{\mathrm{app},i},$$

where *n* is the number of tested specimens (*n* = 5), and *ρ_app_*_,*i*_ is the apparent density of specimen *i*.

The results are reported as mean apparent density ± standard deviation (SD), calculated from the five specimen-level density values. Repeated mass and dimensional measurements were used only to obtain representative values for each individual specimen and were not treated as independent replicates.

#### 2.7.2. Water Absorption and Dimensional Stability

Five specimens were initially tested for formulations 1, 2, 3, 4, and 6, whereas four specimens were available for formulation 7 because an additional specimen could not be prepared. Prior to immersion, the specimens were dried in a BINDER drying oven at 40 °C for 72 h. The temperature of 40 °C was selected as a controlled accelerated water-exposure condition intended to promote water uptake, dimensional changes, and progressive loss of structural integrity within the experimental time frame. The test was therefore designed as a severe comparative screening condition rather than as a direct simulation of a specific service environment. The initial mass (m_0_), thickness (*T*_0_), width (*W*_0_), and length (*L*_0_) of each specimen were determined immediately before immersion.

The initial mass of each specimen was measured three times using a RADWAG AS 220.R2 analytical balance (RADWAG, Radom, Poland), and the arithmetic mean was used as *m*_0_. Before immersion, measurement lines were marked on each specimen to ensure that dimensional measurements were performed at consistent locations throughout the experiment. Thickness, width, and length were measured at three predefined positions using a Mitutoyo Absolute AOS Digimatic CD-15DAX digital caliper (Mitutoyo Corporation, Kanagawa, Japan), and the arithmetic mean of the three measurements was used for further calculations.

Each specimen was placed individually in a closed container and completely immersed in 100 mL of distilled water. The specimens were maintained at 40 °C throughout the experiment, and the water was not replaced during the immersion period. Measurements were performed before immersion (0 h) and after 2, 24, 48, 72, and 168 h of exposure.

At each measurement time, the specimens were removed from the water and gently blotted with a paper towel to remove excess surface water. The wet mass (*m_t_*) was determined immediately using the same analytical balance. Because of rapid water loss from the wet specimen surface, a single mass measurement was performed at each post-immersion time point. Immediately after weighing, specimen thickness, width, and length were measured at the previously marked locations. Each dimension was measured at three predefined positions, and the arithmetic mean was used for further calculations.

For specimens exhibiting strongly non-uniform swelling, quantitative dimensional analysis was based on measurements obtained at the predefined locations used consistently throughout the experiment. Additional local thickness measurements, where recorded to document irregular swelling, were not included in the calculation of specimen-level dimensional changes or formulation-level summary statistics.

Water absorption, *WA_t_*, after immersion time *t* was calculated according to Equation (3):

(3) $${\mathrm{WA}}_{t}=\frac{m_{t}-m_{0}}{m_{0}}\times 100\%$$

where *m_0_* is the initial specimen mass after the 72 h drying step and *m_t_* is the specimen mass after immersion time *t*.

Thickness swelling, *TS_t_*, was calculated according to Equation (4):

(4) $${\mathrm{TS}}_{t}=\frac{T_{t}-T_{0}}{T_{0}}\times 100\%$$

where *T_0_* and *T_t_* are the initial thickness and the thickness after immersion time *t*, respectively.

Width swelling, *WS_t_*, was calculated according to Equation (5):

(5) $${\mathrm{WS}}_{t}=\frac{W_{t}-W_{0}}{W_{0}}\times 100\%$$

where *W_0_* and *W_t_* are the initial width and the width after immersion time *t*, respectively.

Length swelling, *LS_t_*, was calculated according to Equation (6):

(6) $${\mathrm{LS}}_{t}=\frac{L_{t}-L_{0}}{L_{0}}\times 100\%$$

where *L_0_* and *L_t_* are the initial length and the length after immersion time *t*, respectively.

In addition to mass and dimensional measurements, macroscopic appearance and retention of measurable structural integrity were assessed throughout the immersion period. A specimen was considered no longer suitable for quantitative measurement when it separated into several distinct fragments or became sufficiently fragmented that representative mass and dimensional measurements were no longer meaningful. Depending on the specimen, disintegration involved delamination, irregular fragmentation, or complete loss of macroscopic cohesion. If substantial fragments detached from a specimen, no further quantitative measurements were performed for that specimen.

Consequently, the number of measurable specimens decreased during immersion and was recorded separately at each exposure time. Missing values resulting from specimen fragmentation or disintegration were not replaced or imputed.

No post-immersion mechanical testing was performed. Therefore, retention of measurable integrity refers only to the preservation of sufficient macroscopic cohesion for continued mass and dimensional measurements and should not be interpreted as retention of mechanical performance.

#### 2.7.3. Compression Testing

Quasi-static edgewise compression tests were performed using two testing systems depending on the required force range. An MTS Insight universal testing machine (MTS Systems Corporation, Eden Prairie, MN, USA) equipped with a ±1 kN load cell was used for specimens within this force range. Specimens requiring a higher force range were tested using an MTS 858 Table Top System (MTS Systems Corporation, Eden Prairie, MN, USA) with a ±25 kN force-measurement range.

Five specimens were tested for each formulation (*n* = 5) with nominal dimensions of 29 × 29 × 5 mm. The original 29 × 29 mm plate face was oriented vertically between the compression platens, and the compressive load was applied along one of the approximately 29 mm in-plane directions of the original composite plate. Accordingly, the initial load-bearing cross-section used for stress calculation was approximately 29 × 5 mm^2^. The actual measured specimen dimensions were used to calculate the initial edgewise load-bearing cross-sectional area for each individual specimen rather than the nominal dimensions. The crosshead speed was 1 mm × min^−1^, and no initial preload was applied. Loading was continued until pronounced structural failure or loss of specimen integrity, identified by a distinct decrease in the recorded compressive force. Force and crosshead displacement were continuously recorded.

The highest compressive force recorded among all tested specimens was approximately 1.24 kN and was obtained using the higher-capacity MTS 858 system. All recorded peak forces remained within the measurement range of the system used for the respective test.

Compressive stress was calculated from the recorded force using the initial edgewise load-bearing cross-sectional area. Compressive stresses are reported as positive magnitudes; therefore, the maximum compressive stress was defined as the absolute value of the minimum recorded compressive stress.

#### 2.7.4. Three-Point Flexural Testing

Flexural properties of the mycelium composites were determined by static three-point bending using an MTS Insight universal testing machine (MTS Systems Corporation, Eden Prairie, MN, USA), according to ISO 178 [30]. Rectangular specimens with nominal in-plane dimensions of approximately 80 × 10 mm were tested using a support span of 60 mm and a crosshead speed of 2 mm × min^−1^. The actual thickness of each specimen was measured individually before testing and ranged from 4.09 to 6.06 mm across the investigated specimens. No through-thickness machining, sanding, or planing was performed after hot pressing; therefore, the original upper and lower plate surfaces were preserved. Ten specimens were analyzed for each material formulation (*n* = 10).

The specimens were positioned centrally on two supports and loaded at the midpoint of the support span. For all formulations, specimen orientation relative to the original plate surfaces was maintained consistently. The surface that had faced the metal tray during hot pressing, designated as the lower surface, was positioned downward during three-point bending and therefore constituted the tensile face. The upper surface was located on the compressive side. No initial preload was applied. Loading was continued until specimen failure or loss of structural integrity. The load-cell capacity was ±1 kN, with a reading noise of approximately ±0.05 N. The loading nose and support rollers had a radius of 5 mm.

The maximum flexural stress, σ_fM_, was calculated from the maximum force recorded during the test according to Equation (7):

(7) $$\sigma_{f}=\frac{3\mathrm{FL}}{{2\mathrm{bh}}^{2}}$$

where *F* is the maximum applied force [N], *L* is the support span [mm], *b* is the specimen width [mm], and *h* is the specimen thickness [mm]. The individually measured specimen width and thickness were used in the calculation for each specimen rather than nominal dimensions.

Flexural strain, ɛ_f_, was calculated according to Equation (8):

(8) $$\varepsilon_{f}=\frac{6\mathrm{sh}}{L^{2}}$$

where *s* is the mid-span deflection [mm], *h* is the specimen thickness [mm], and *L* is the support span [mm]. In the present study, the crosshead displacement recorded by the testing machine was used as an approximation of the mid-span deflection. No correction for testing-machine compliance was applied.

The flexural modulus, *E_f_*, was determined from the approximately linear portion of the flexural stress–strain response within the strain range of 0.0005–0.0025 and was calculated according to Equation (9):

(9) $$E_{f}=\frac{\sigma_{f,2}-\sigma_{f,1}}{\varepsilon_{f,2}-\varepsilon_{f,1}}$$

where σ_f,1_ and σ_f,2_ are the flexural stresses corresponding to flexural strains ε_f,1_ = 0.0005 and ε_f,2_ = 0.0025, respectively. The same strain interval was used for all specimens.

#### 2.7.5. Statistical Analysis

Quantitative results are presented as mean ± standard deviation (SD), together with individual specimen values where appropriate. Five specimens were analyzed for apparent density and compression testing, whereas ten specimens were tested in three-point bending for each formulation. Individual measurements were retained unless an objective experimental or measurement-related reason for exclusion was identified.

The association between apparent density and maximum compressive stress was evaluated descriptively using Spearman’s rank correlation coefficient (ρ) based on 30 paired specimen-level observations pooled across the six investigated plates. To examine whether the pooled pattern persisted after removal of formulation-level mean differences, a secondary within-formulation-centered analysis was performed. For each specimen, apparent density and maximum compressive stress were centered by subtracting the corresponding formulation mean, and Spearman’s rank correlation coefficient was calculated between the centered variables. Formulation-specific Spearman coefficients were also calculated descriptively. Because all observations represent within-plate subsamples from one manufactured plate per formulation, no formal significance testing was applied to these correlations. The coefficients characterize specimen-level covariation within the investigated plates and should not be interpreted as population-level estimates or independent tests of formulation effects.

Water-absorption and dimensional-change data were analyzed descriptively because progressive specimen fragmentation reduced the number of specimens remaining measurable during immersion. Results are reported as mean ± SD together with the number of measurable specimens at each exposure time; when only one specimen remained, the individual value is reported without SD. Missing values resulting from specimen disintegration were not imputed.

Statistical analyses were performed using OriginPro 2021 (OriginLab Corporation, Northampton, MA, USA). All specimen-level observations originated from one manufactured plate per formulation and therefore characterize within-plate variability rather than manufacturing-replicate variability.

## 3. Results

### 3.1. Visual Appearance and Macroscopic Structure

**Figure 2:**
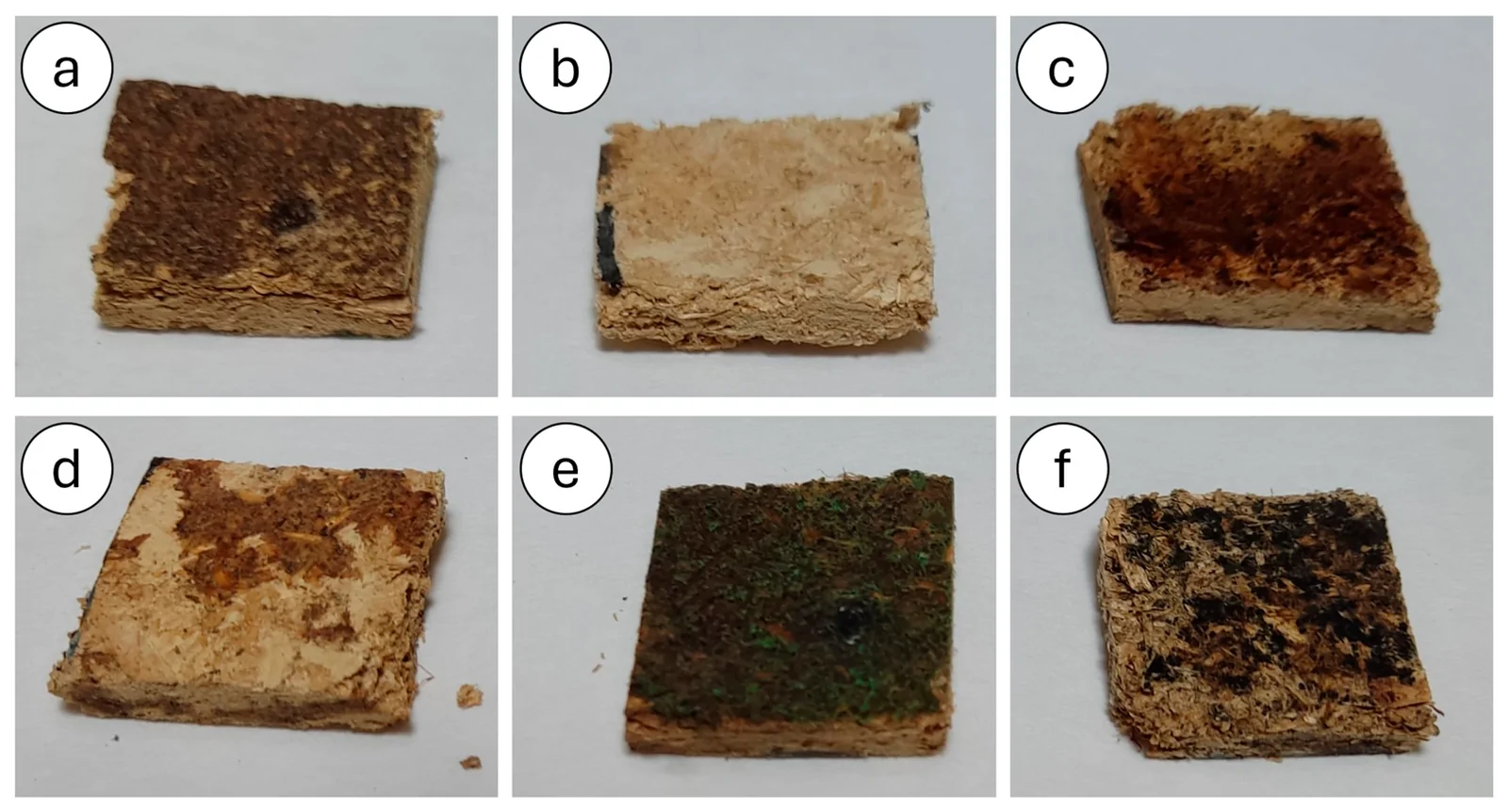
Representative as-prepared mycelium composite specimens prior to physical and mechanical testing: (**a**) formulation 1; (**b**) formulation 2; (**c**) formulation 3; (**d**) formulation 4; (**e**) formulation 6; and (**f**) formulation 7.

Representative as-prepared specimens of the six investigated mycelium composite formulations are shown in Figure 2. All formulations produced cohesive plates after cultivation, drying, and hot pressing, although clear differences in surface appearance and degree of consolidation were observed among the materials.

The composite surfaces showed heterogeneous morphology, with local differences in texture and visibility of the lignocellulosic constituents. Cross-sectional observations also revealed through-thickness variability, indicating that hot pressing consolidated the material while preserving local structural heterogeneity. Differences between the upper and lower surfaces were particularly evident in formulations in which PCL had initially been introduced in mesh form. In formulation 1, PCL meshes had initially been positioned at both outer surfaces, whereas in formulation 6 a single mesh had initially been positioned at the lower surface. The present macroscopic observations do not establish preservation of the original printed mesh geometry after hot pressing.

### 3.2. Apparent Density

**Figure 3:**
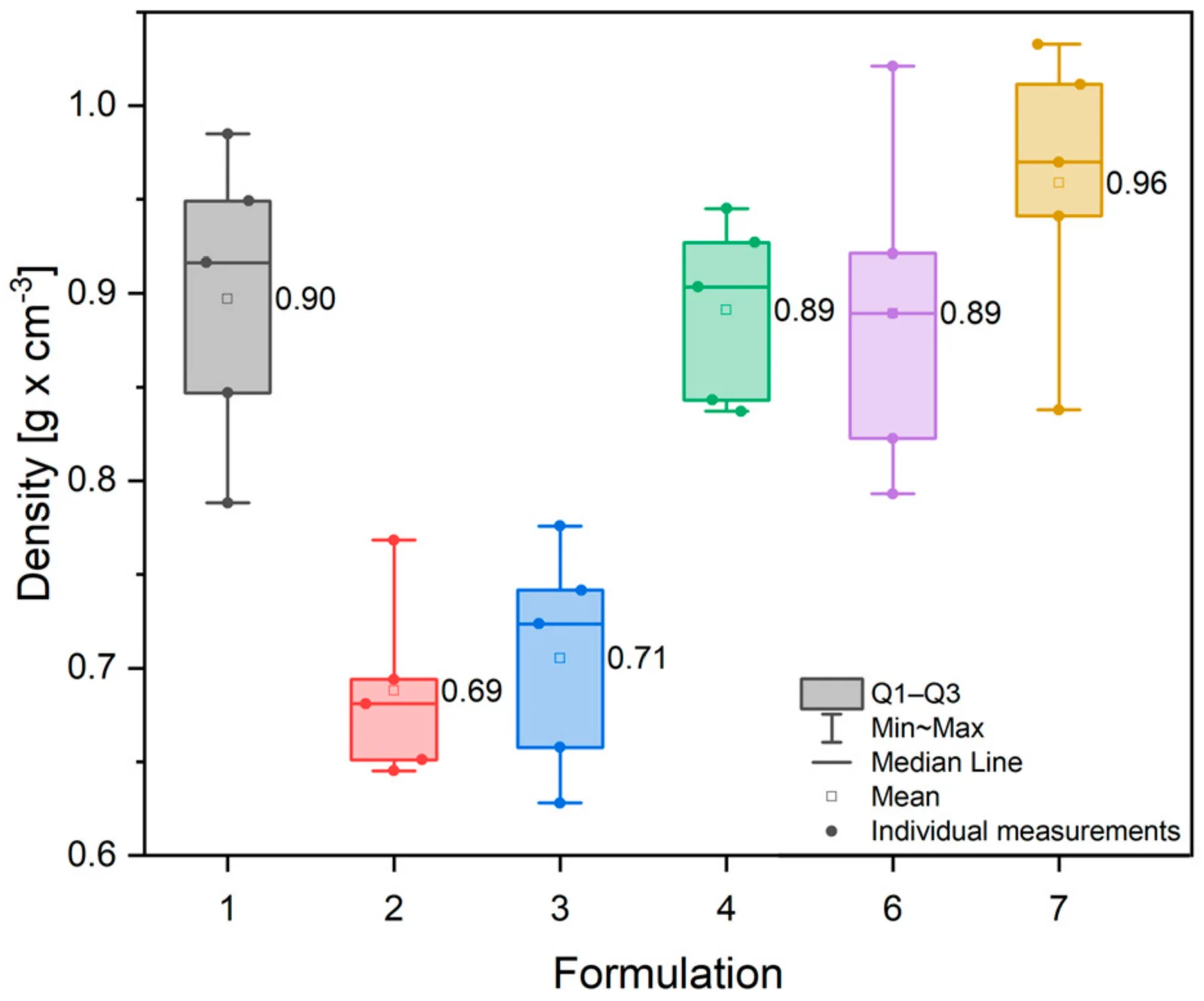
Apparent density of the investigated mycelium composite formulations. Individual specimen-level values (*n* = 5 per formulation) are shown together with box-and-whisker plots. Boxes indicate the interquartile range (Q1–Q3), whiskers indicate the minimum and maximum values, the horizontal line represents the median, and the mean is shown separately.

The apparent density, ρ_ap p_, of the investigated mycelium composites ranged from approximately 0.69 to 0.96 g *×* cm^−3^ (Figure 3). Formulations 2 and 3 occupied the lower-density range, whereas formulations 1, 4, 6, and 7 exhibited generally higher apparent densities. The highest mean apparent density was observed for formulation 7.

Inspection of the individual specimen values revealed considerable within-formulation variability. This was particularly evident for formulation 6, which exhibited the broadest range of individual density values (0.793–1.021 g *×* cm^−3^), despite having a mean apparent density similar to those of formulations 1 and 4. Thus, comparable formulation-level mean densities could correspond to substantially different specimen-level density distributions.

### 3.3. Water Absorption and Dimensional Response

Water immersion resulted in pronounced formulation-dependent differences in water uptake, dimensional changes, and retention of measurable specimen integrity. Formulations 1 and 7 lost measurable integrity before the first scheduled measurement at 2 h. Three of the five specimens of formulation 2 remained measurable throughout the 168 h exposure period. In formulation 3, two specimens remained measurable after 2 h and only one from 24 h onward. Four specimens of formulation 4 remained measurable up to 72 h, whereas none retained sufficient integrity for quantitative measurement after 168 h. Formulation 6 showed the greatest retention of measurable integrity, with all five specimens remaining measurable up to 72 h and four remaining measurable after 168 h.

**Figure 4:**
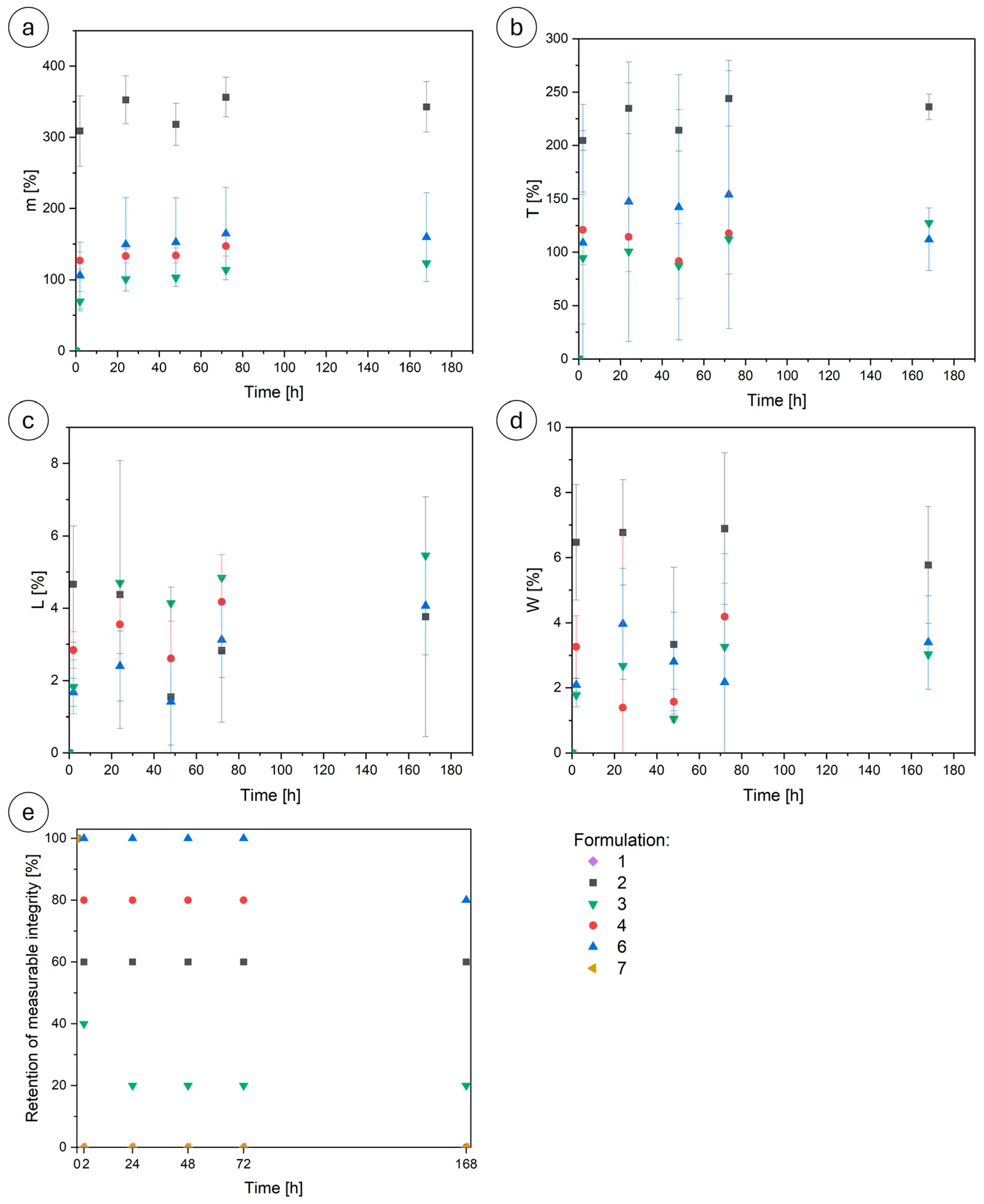
Water-induced changes in the investigated mycelium composites as a function of immersion time: (**a**) water absorption, (**b**) thickness swelling, (**c**) length swelling, (**d**) width swelling, (**e**) retention of measurable integrity as a function of immersion time. Panels (**a**–**d**) show quantitative measurements only for specimens that remained measurable at the respective observation time and therefore represent survivor-conditioned data. Panel (**e**) shows the percentage of the initial specimen population that retained sufficient macroscopic integrity for continued quantitative measurement at each scheduled observation time. Observation times are plotted at the actual elapsed times of 0, 2, 24, 48, 72, and 168 h. Markers in panel E are not connected because the exact time of integrity loss between scheduled observations was not determined. Results are presented as mean ± standard deviation for specimens remaining measurable at each exposure time. The numbers of measurable specimens at 2, 24, 48, 72, and 168 h were, respectively: formulation 2, *n* = 3/3/3/3/3; formulation 3, *n* = 2/1/1/1/1; formulation 4, *n* = 4/4/4/4/0; and formulation 6, *n* = 5/5/5/5/4. No post-immersion measurements were possible for formulations 1 and 7. For *n* = 1, individual values are shown without a standard-deviation error bar.

Water absorption and dimensional changes in the specimens remaining measurable at each exposure time are shown in Figure 4. Formulation 2 exhibited the highest water uptake, increasing from 309 ± 49% after 2 h to a maximum mean value of 356 ± 28% after 72 h and remaining high after 168 h (343 ± 35%). Formulations 3, 4, and 6 showed lower water absorption. Formulation 4 increased from 127 ± 13% after 2 h to 147 ± 14% after 72 h, whereas formulation 6 reached a maximum mean value of 165 ± 65% after 72 h. For formulation 3, only one specimen remained measurable from 24 h onward; consequently, later values represent the response of an individual specimen rather than a formulation-level mean.

Water-induced dimensional changes occurred predominantly in the through-thickness direction. Formulation 2 exhibited the greatest mean thickness swelling, increasing from 205 ± 9% after 2 h to a maximum of 244 ± 26% after 72 h. Formulation 6 showed lower mean thickness swelling but substantially greater specimen-to-specimen variability, with standard deviations exceeding 120% at several exposure times. Formulations 3 and 4 exhibited intermediate responses. In contrast, in-plane dimensional changes were limited, with width and length changes generally remaining below approximately 7%.

**Figure 5:**
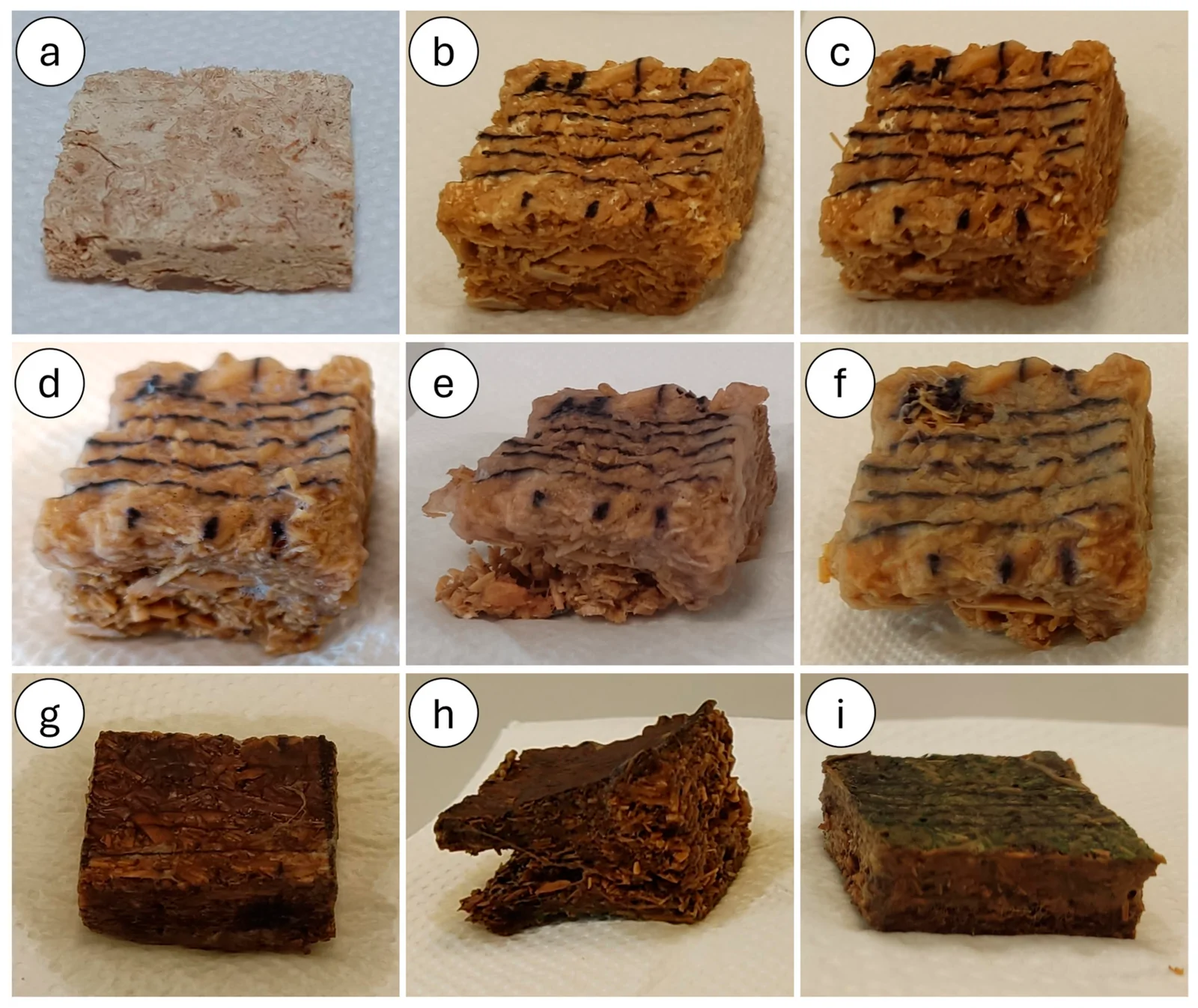
Macroscopic observations of the formulations during water immersion: (**a**–**f**) formulation 2 at different immersion times: (**a**) before immersion; (**b**) 2 h; (**c**) 24 h; (**d**) 48 h; (**e**) 72 h; and (**f**) 168 h; (**g**) formulation 3 retaining measurable integrity after 168 h; (**h**) formulation 4 after 168 h, showing loss of measurable integrity; (**i**) formulation 6 retaining measurable integrity after 168 h.

Progressive swelling, deformation, and loss of macroscopic cohesion were also observed during immersion. Representative morphological changes in formulation 2 are shown in Figure 5a–f. After 168 h, selected specimens of formulations 3 and 6 retained sufficient integrity for quantitative measurement, whereas formulation 4 no longer retained measurable integrity (Figure 5g–i).

Overall, water uptake, dimensional swelling, and retention of measurable structural integrity did not follow a single common pattern. Formulation 2 combined the highest water absorption and through-thickness swelling with retention of three measurable specimens after 168 h, whereas formulation 6 retained the greatest proportion of measurable specimens despite pronounced and heterogeneous swelling. In contrast, formulations 1 and 7 lost measurable integrity before the first post-immersion measurement. Thus, the magnitude of water uptake or dimensional swelling alone did not determine the ability of the composites to retain macroscopic cohesion during prolonged immersion.

### 3.4. Compressive Response

**Figure 6:**
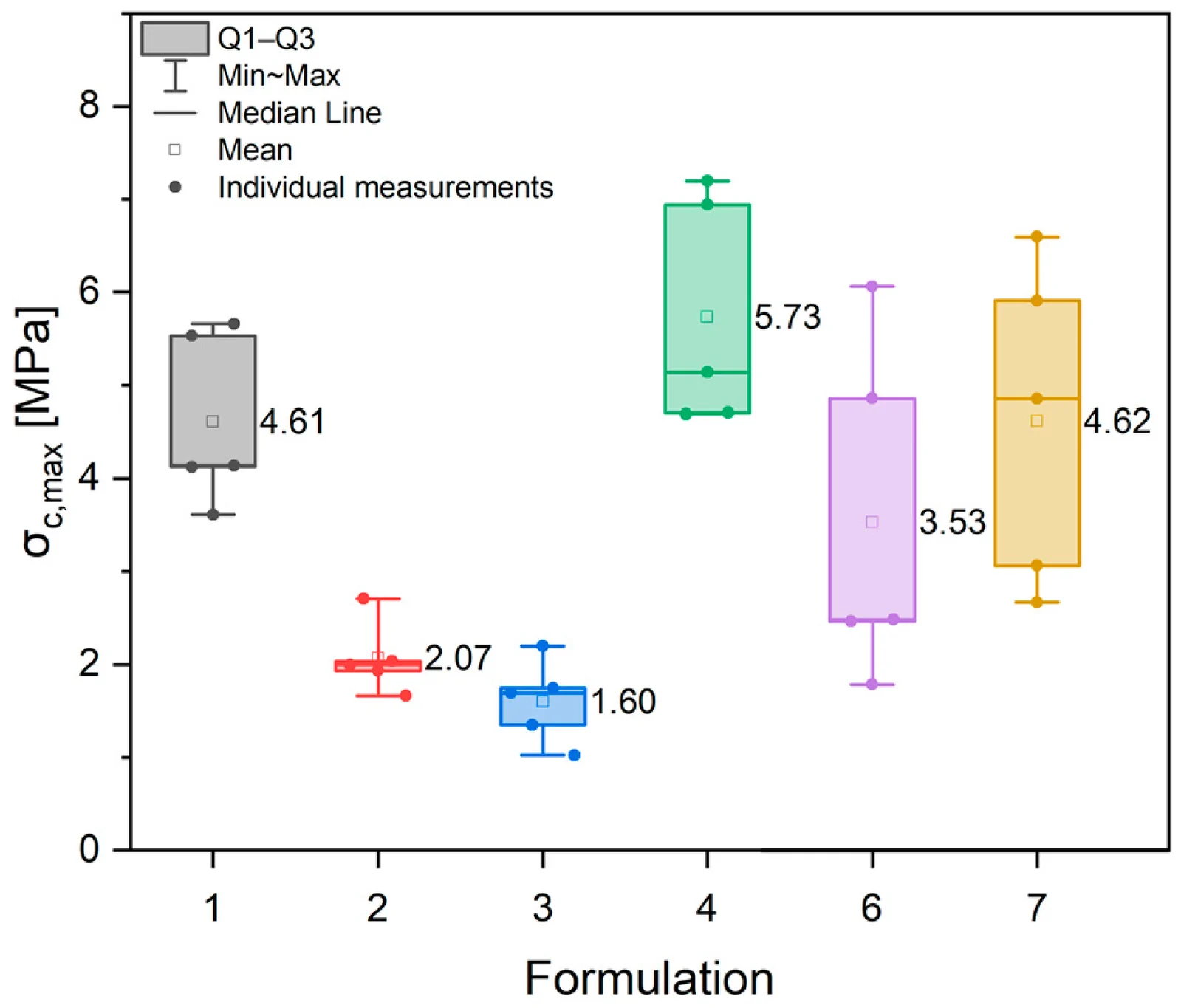
Maximum compressive stress of the investigated mycelium composite formulations. Individual specimen-level values (*n* = 5 per formulation) are shown together with box-and-whisker plots. Boxes indicate the interquartile range (Q1–Q3), whiskers indicate the minimum and maximum values, the horizontal line represents the median, and the mean is shown separately.

The compressive response differed markedly among the investigated mycelium composite formulations (Figure 6). The mean maximum compressive stress, *σ*_c,max_, ranged from 1.6 ± 0.4 MPa for formulation 3 to 5.7 ± 1.2 MPa for formulation 4. Formulations 1 and 7 also exhibited relatively high mean values of approximately 4.6 MPa, whereas formulation 6 showed an intermediate response (3.5 ± 1.8 MPa). Formulations 2 and 3 exhibited the lowest mean maximum compressive stresses.

Individual specimen values revealed substantial within-formulation variability, particularly for formulations 1, 4, 6, and 7. The widest range was observed for formulation 6, where maximum compressive stress varied from approximately 1.8 to 6.1 MPa. Several formulations showed an apparent separation into lower- and higher-response groups rather than a compact distribution around the mean. Because only five specimens were tested per formulation, this pattern is interpreted descriptively as apparent clustering and not as statistically confirmed bimodality.

### 3.5. Flexural Properties

**Figure 7:**
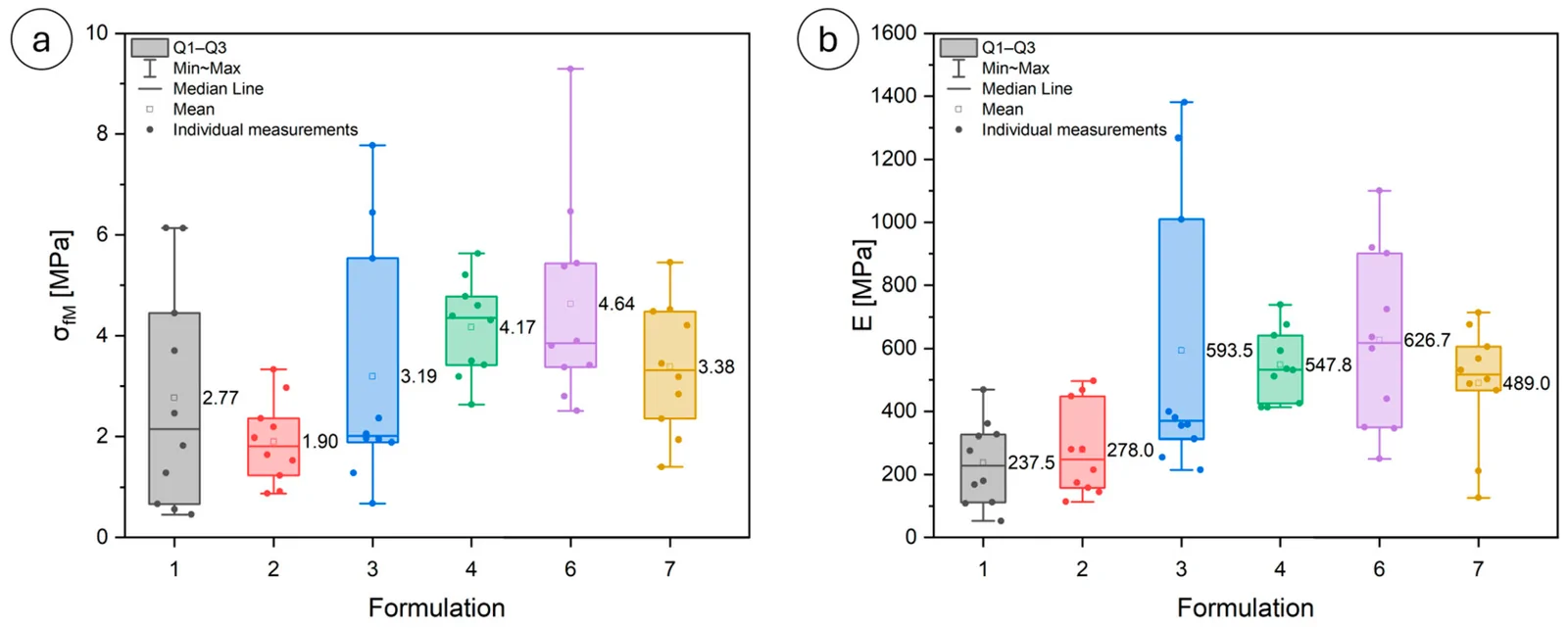
Flexural properties of the investigated mycelium composite formulations: (**a**) maximum flexural stress and (**b**) flexural modulus. Individual specimen-level values (*n =* 10 per formulation) are shown together with box-and-whisker plots. Boxes indicate the interquartile range (Q1–Q3), whiskers indicate the minimum and maximum values, the horizontal line represents the median, and the mean is shown separately.

The flexural response differed considerably among the investigated mycelium-composite formulations and was accompanied by substantial specimen-to-specimen variability (Figure 7). Mean maximum flexural stress ranged from 1.9 ± 0.8 MPa for formulation 2 to 4.6 ± 2.1 MPa for formulation 6. Formulation 4 also exhibited a relatively high mean maximum flexural stress (4.2 ± 1.0 MPa), whereas formulations 1, 2, 3, and 7 showed lower mean values.

A similar formulation-dependent pattern was observed for the flexural modulus. The highest mean value was obtained for formulation 6 (627 ± 285 MPa), followed by formulation 3 (594 ± 445 MPa) and formulation 4 (548 ± 114 MPa). The large standard deviations for formulations 3 and 6 reflected pronounced differences among individual specimens.

Inspection of the specimen-level data revealed apparent lower- and higher-response groups in several formulations, most clearly in formulation 3. Formulation 6 also exhibited a broad range of flexural responses, whereas formulation 4 showed a comparatively narrower distribution. Because these patterns were observed in finite specimen populations, they are described as apparent clustering rather than as statistically confirmed bimodal distributions.

Macroscopic examination after three-point bending revealed localized damage in the loading region. Representative post-bending damage morphology is provided in Supplementary Figure S2.

### 3.6. Cross-Comparison of Physical, Water-Exposure, and Mechanical Response

Comparison of the investigated properties revealed distinct formulation-specific performance profiles (Table 3). Similar mean apparent densities were not necessarily associated with similar mechanical or water-exposure behavior. Formulations 1, 4, 6, and 7 exhibited comparable mean apparent densities of approximately 0.89–0.96 g cm^−3^, yet differed markedly in compressive response, flexural properties, and retention of measurable integrity during immersion.

**Figure 8:**
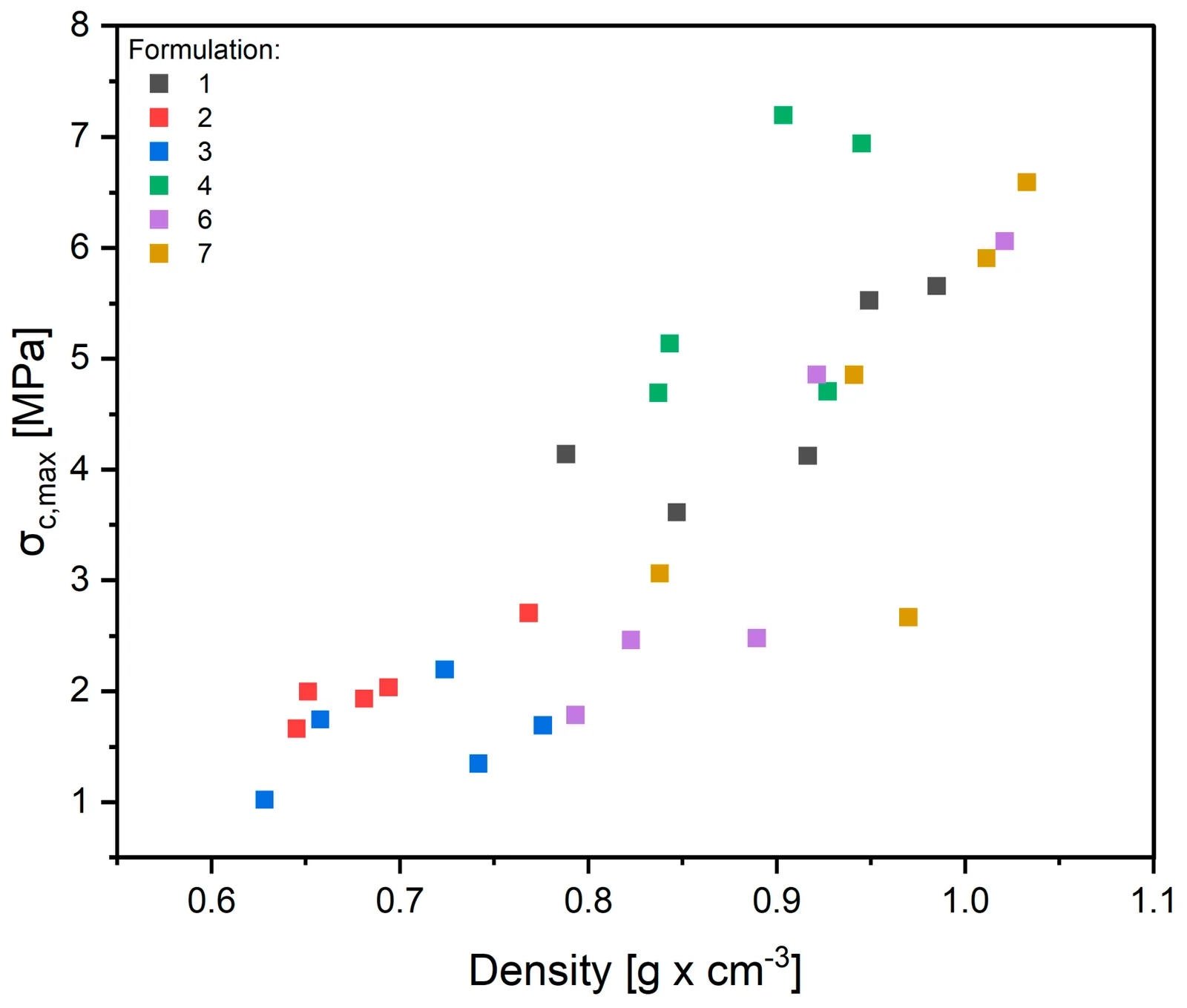
Relationship between apparent density and maximum compressive stress of the investigated mycelium-composite specimens. Each point represents an individual specimen for which apparent density and compressive response were determined on the same physical specimen (*n* = 30). Different colors indicate the respective formulations. No regression model is superimposed; statistical associations were evaluated using Spearman’s rank correlation as described in Section 2.7.5.

Because apparent density and compressive response were determined for the same individual specimens, their relationship was evaluated at the specimen level (Figure 8). The pooled analysis showed a positive association between apparent density and maximum compressive stress (Spearman’s ρ = 0.845, *n* = 30). A positive association was also observed after within-formulation centering (ρ = 0.713, *n* = 30). Formulation-specific Spearman coefficients were positive for all six investigated plates, although their magnitude varied (formulations 1, 2, 3, 4, 6, and 7: ρ = 0.70, 0.90, 0.20, 0.50, 1.00, and 0.70, respectively). These coefficients are interpreted descriptively because each formulation was represented by within-plate subsamples obtained from a single manufactured plate.

Cross-comparison of the mechanical and water-exposure results further showed that favorable performance in one property did not necessarily correspond to favorable performance in another. Formulation 4 exhibited the highest mean maximum compressive stress, whereas formulation 6 showed the highest mean maximum flexural stress and flexural modulus and retained the greatest proportion of measurable specimens after 168 h of immersion. In contrast, formulation 2 combined relatively low dry mechanical properties with the highest water absorption and through-thickness swelling, yet three of five specimens remained measurable after 168 h. Formulations 1 and 7 lost measurable integrity before the first post-immersion measurement despite belonging to the higher-density group.

Overall, no single formulation was superior across all evaluated properties. The results therefore indicate formulation-specific trade-offs among apparent density, dry mechanical performance, water uptake, dimensional response, and retention of measurable integrity.

## 4. Discussion

### 4.1. Formulation-Dependent Mechanical Response and Structural Heterogeneity

The investigated mycelium composites showed clearly formulation-dependent mechanical behavior. Formulation 4 exhibited the highest mean maximum compressive stress, whereas formulation 6 showed the highest mean maximum flexural stress and flexural modulus. This divergence suggests that the measured compression and flexural responses were sensitive to different aspects of the heterogeneous composite structure. However, the present data do not identify the specific structural mechanisms responsible for these differences. Such behavior is consistent with the heterogeneous and porous nature of mycelium-based materials, in which compression is influenced by local densification and collapse of porous regions, whereas flexural response is additionally sensitive to tensile-side defects, through-thickness continuity, and local weak zones [7,8,9,17].

In formulation 6, PCL had initially been introduced at the lower plate surface, which was subsequently positioned on the tensile side during three-point bending. This configuration may be relevant to the comparatively high flexural response of this material; however, because the post-pressing PCL morphology was not quantitatively characterized and the formulation differed simultaneously in several other variables, an independent reinforcement effect cannot be established.

Apparent density was positively associated with maximum compressive stress both in the pooled specimen-level analysis and after within-formulation centering. The persistence of the association after removal of formulation-level mean differences indicates that the pooled relationship was not driven solely by the lower mean densities and compressive responses of formulations 2 and 3 relative to the remaining materials. However, the magnitude of the within-formulation associations varied considerably, and each coefficient was based on only five subsamples originating from a single manufactured plate. The relationship should therefore be interpreted as a specimen-level association within the investigated plates rather than as evidence of a population-level formulation effect.

Considerable specimen-to-specimen variability was observed in both compression and bending, with several formulations showing apparent lower- and higher-response groups. Similar variability has been attributed to the intrinsically heterogeneous architecture of mycelium composites, including non-uniform substrate packing, local differences in colonization, pore distribution, consolidation, and reinforcement distribution [2,7,8,17,20].

In the present study, the macroscopic observations of the hot-pressed plates were consistent with this interpretation, as local differences in surface and through-thickness structure were evident.

Nevertheless, the observed clustering should not be attributed directly to different degrees of fungal colonization. The available data do not allow the relative contributions of mycelial distribution, local compaction, polymeric-phase arrangement, or internal defects to be separated. Therefore, the lower- and higher-response groups are more appropriately interpreted as a manifestation of specimen-scale structural heterogeneity rather than as evidence of two discrete material populations. This also supports the presentation of individual specimen values alongside mean ± standard deviation, because removal of mechanically high- or low-response specimens without an objective experimental reason could eliminate physically meaningful information from these heterogeneous bio-based materials.

### 4.2. Water-Exposure Behavior, Anisotropic Swelling, and Structural Integrity

Water immersion revealed pronounced formulation-dependent differences that were not explained by apparent density or dry-state mechanical properties alone. The results indicate that water uptake, dimensional deformation, and retention of measurable structural integrity should be treated as related but distinct descriptors of moisture response in mycelium composites [8,18,19,20].

Formulation 2 provides a clear example of this distinction. Despite exhibiting the highest water absorption and the greatest mean through-thickness swelling, three of the five initial specimens remained measurable after 168 h. Formulation 6 showed a different response: it retained the greatest proportion of measurable specimens after prolonged immersion, although its thickness swelling was highly heterogeneous. These observations show that extensive water uptake or dimensional expansion does not necessarily result in immediate loss of macroscopic cohesion.

The dimensional response was strongly anisotropic, with through-thickness swelling substantially exceeding changes in width and length. This behavior is consistent with the plate-like geometry and prior hot-pressing of the composites, which promote preferential recovery and expansion in the thickness direction during water exposure [8,17,23]. Because the present study did not include quantitative internal-structure characterization during immersion, the specific contributions of pore architecture, local consolidation, and mycelial distribution to this anisotropy cannot be resolved.

In contrast, formulations 1 and 7 lost measurable integrity before the first scheduled post-immersion measurement, despite belonging to the higher-density group and showing relatively favorable dry-state mechanical properties. This confirms that apparent density and dry mechanical resistance cannot be used as direct indicators of water-exposure stability.

Interpretation of late-time measurements also requires consideration of specimen loss. In formulation 3, only one specimen remained measurable from 24 h onward; consequently, later values represent the response of a surviving specimen rather than the original formulation population. Continued measurability should therefore be interpreted as retention of sufficient cohesion for quantitative assessment, not as preservation of the initial specimen morphology.

Overall, the results support the use of water absorption together with dimensional change and specimen-integrity retention when evaluating moisture-sensitive mycelium composites. Water uptake alone does not capture the full wet-state response, and late-time measurements should always be considered together with the number of specimens remaining measurable [8,18,19,20,21].

### 4.3. Cross-Property Trade-Offs and the Role of Polymeric-Phase Architecture

Cross-comparison of the investigated properties showed that no single formulation was superior in all performance categories. Formulation 4 combined the highest compressive resistance with relatively high flexural properties, whereas formulation 6 exhibited the highest mean maximum flexural stress and flexural modulus together with the greatest retention of measurable integrity during prolonged immersion. In contrast, formulation 2 showed relatively low dry-state mechanical properties but retained measurable cohesion in three of five specimens after 168 h despite the highest water uptake and through-thickness swelling. These contrasting responses suggest that mechanical performance, dimensional stability, and wet-state integrity may be sensitive to different aspects of the composite structure; however, the specific structural mechanisms underlying these differences remain unresolved.

The initially introduced physical form and spatial arrangement of the polymeric component represent potentially relevant variables for future controlled investigation. In the present materials, polymeric components were introduced as dispersed granules, dispersed film flakes, or localized PCL meshes. However, the morphology retained by these phases after hot pressing was not quantitatively characterized.

Formulation 6, in which PCL had initially been introduced as a mesh at the lower surface, retained the greatest proportion of measurable specimens after 168 h and exhibited the highest mean flexural response. These observations are hypothesis-generating rather than evidence of a direct effect of polymer configuration. Formulation 1 also contained initially introduced PCL meshes but lost measurable integrity before the first post-immersion measurement, while formulations 4 and 7 both contained initially dispersed PCL granules but exhibited markedly different responses. Because fungal species, starch component, substrate composition, and polymer configuration changed simultaneously among the investigated materials, the independent contribution of polymer architecture cannot be isolated from the present dataset.

Apparent density also showed property-specific predictive value. Although apparent density showed a positive specimen-level association with maximum compressive stress, formulations with comparable mean densities still differed substantially in flexural behavior and water-exposure stability. Density therefore reflects the overall degree of consolidation but does not fully describe the local architecture governing deformation, damage, and loss of cohesion [7,8,9,17].

These results indicate that optimization of mycelium composites should focus on balancing loading-mode-specific mechanical performance with dimensional and wet-state stability rather than maximizing a single parameter. Polymeric-phase architecture appears to be a relevant design variable, but its independent contribution should be verified using controlled formulations in which polymer geometry and spatial arrangement are varied while the remaining compositional factors are kept constant.

### 4.4. Limitations and Implications for Material Design

Several limitations should be considered when interpreting the present results. Only one plate was produced for each formulation. Consequently, the specimen populations characterize within-plate heterogeneity and cannot be used to quantify plate-to-plate or batch-to-batch manufacturing reproducibility. Formulation-level comparisons should therefore be interpreted as exploratory comparisons of the manufactured material systems rather than population-level estimates of formulation effects. Because the hot-pressing process was controlled by final platen separation rather than by prescribed force or pressure, and the applied force was not recorded, the consolidation history cannot be quantitatively reproduced on the basis of pressure–time data.

The investigated formulations differed simultaneously in fungal species, starch-based component, spent coffee grounds, and polymeric-phase architecture. The study therefore compares complete material systems rather than independently varied constituent effects. Consequently, differences between formulations cannot be attributed to any single factor, including fungal species or polymeric-phase geometry.

The relatively small specimen populations, particularly in compression, limit formal characterization of the apparent lower- and higher-response groups. Although the observed clustering is consistent with the heterogeneous structure reported for mycelium-based composites, larger datasets would be required to establish whether these patterns represent statistically distinct subpopulations rather than continuous material variability [2,8,17,20].

The present macroscopic observations do not quantitatively resolve internal porosity, local mycelial distribution, polymer location, interfacial quality, or through-thickness gradients in consolidation. These structural features may contribute to the pronounced specimen-to-specimen variability observed in both mechanical and water-exposure behavior [7,8,17]. Future studies should therefore combine mechanical and immersion testing with quantitative internal-structure characterization, for example by X-ray computed tomography or spatially resolved microscopy.

The mechanical-test configuration introduces additional limitations. Because the compression specimens were tested edgewise, possible contributions from specimen instability or local buckling cannot be completely excluded. The reported maximum compressive stresses should therefore be interpreted as directional in-plane compressive responses under the applied test configuration rather than as orientation-independent compressive strength values. In three-point bending, flexural strain was derived from crosshead displacement used as an approximation of mid-span deflection, and no correction for testing-machine compliance was applied. Accordingly, the reported E_f_ values should be interpreted as apparent flexural moduli obtained under the applied test configuration.

Interpretation of the immersion results is additionally affected by progressive specimen fragmentation. Because specimen loss formed part of the material response itself, the number of measurable specimens decreased with exposure time and differed among formulations. Late-time water-absorption and swelling values should therefore be interpreted together with the number of specimens remaining measurable rather than as directly equivalent formulation-level averages. Moreover, prolonged immersion in liquid water represents a severe exposure condition and should not be considered a complete surrogate for service-specific humidity or cyclic environmental conditions [8,18,21].

Despite these limitations, the results identify several parameters that should be considered simultaneously in future mycelium composite design: loading-mode-specific mechanical performance, specimen-to-specimen variability, dimensional response to moisture, and retention of wet-state structural integrity. The polymeric phase appears to be a potentially important design variable, but its independent role should be evaluated using controlled formulations in which polymer geometry and spatial arrangement are varied while fungal species, substrate composition, and processing conditions are kept constant [24,25,26].

## 5. Conclusions

The investigated hemp-based mycelium composites exhibited distinct formulation-specific combinations of mechanical performance and water-exposure behavior. Because only one plate was manufactured per formulation, the formulation-specific comparisons reported here should be interpreted as exploratory observations of the investigated manufactured plates and their within-plate heterogeneity rather than as estimates of plate-to-plate reproducibility or population-level formulation effects. Apparent density showed a positive specimen-level association with maximum compressive stress, but density alone did not fully explain the mechanical response or predict wet-state stability. Formulation 4 showed the highest mean maximum compressive stress, whereas formulation 6 exhibited the highest mean maximum flexural stress and flexural modulus together with the greatest retention of measurable integrity after prolonged immersion.

Water uptake, dimensional swelling, and retention of macroscopic cohesion represented related but distinct aspects of material performance. Formulation 2 showed the highest water absorption and through-thickness swelling, yet three of five specimens remained measurable after 168 h, whereas formulations 1 and 7 lost measurable integrity before the first post-immersion measurement. These results demonstrate that extensive water uptake or swelling does not necessarily lead directly to rapid structural disintegration.

Overall, no single formulation was superior across all evaluated properties. The results highlight the need to balance loading-mode-specific mechanical performance, structural heterogeneity, dimensional response, and wet-state integrity when designing mycelium-based composites. The observed differences further suggest that the physical form and spatial arrangement of the polymeric phase may contribute to the resulting material response. However, because several compositional and structural variables differed simultaneously among the investigated materials, the independent effects of fungal species, substrate composition, and polymeric-phase architecture cannot be clearly separated in the present study. Further studies using controlled formulations differing in one variable at a time, particularly in the form and distribution of the polymeric phase, are therefore needed to clarify these effects.

## Figures and Tables

**Table 1:** Qualitative composition and initially introduced polymeric-phase configuration of the investigated mycelium composites.

Formulation	Fungal Species	Lignocellulosic Substrate	Additional Bio-Based Component	Starch-Based Component	Initially Introduced Polymeric-Phase Configuration
1	*P. ostreatus*	hemp hurds + hemp straw	spent coffee grounds	TPS	PCL meshes at the outer surfaces
2	*P. ostreatus*	hemp hurds + hemp straw	-	plasticized starch	dispersed polymer film flakes
3	*P. ostreatus*	hemp hurds + hemp straw	spent coffee grounds	plasticized starch	dispersed polymer film flakes
4	*P. ostreatus*	hemp hurds + hemp straw	spent coffee grounds	TPS	dispersed PCL granules
5	*G. applanatum*	hemp hurds + hemp straw	spent coffee grounds	plasticized starch	dispersed polymer film flakes
6	*G. applanatum*	hemp hurds + hemp straw	spent coffee grounds	plasticized starch	PCL mesh at the lower surface
7	*G. applanatum*	hemp hurds + hemp straw	spent coffee grounds	plasticized starch	dispersed PCL granules

* Formulation 5 was not included in the quantitative characterization because sufficient specimen integrity for reproducible testing could not be achieved.

**Table 2:** Quantitative composition of the investigated mycelium-composite formulations per mold.

Formulation	Fungal Species	Hemp Hurds [g]	Hemp Straw [g]	Spent Coffee Grounds [g]	TPS [g]	Plasticized Starch [g]	Polymeric Component
1	*P. ostreatus*	120	30	50	50	–	2 PCL meshes, positioned at the top and bottom surfaces
2	*P. ostreatus*	120	30	–	–	30	45 g polymer film flakes
3	*P. ostreatus*	120	30	30	–	30	45 g polymer film flakes
4	*P. ostreatus*	120	30	50	50	–	40 g PCL granules
5	*G. applanatum*	120	30	50	–	45	20 g polymer film flakes
6	*G. applanatum*	150	40	40	–	40	1 PCL mesh, positioned at the bottom surface
7	*G. applanatum*	150	40	40	–	40	20 g PCL granules

* Formulation 5 was not included in the quantitative characterization because sufficient specimen integrity for reproducible testing could not be achieved. The mass of the printed PCL meshes used in formulations 1 and 6 was not recorded during fabrication; therefore, only the number and initial position of the meshes are reported.

**Table 3:** Cross-comparison of apparent density, mechanical properties, and water-exposure outcome of the investigated mycelium composite formulations. Density and mechanical properties are reported as mean ± standard deviation.

Formulation	ρ_app_ [g *×* cm^−3^]	σ_c,max_ [MPa]	σ_fM_ [MPa]	E_f_ [MPa]	Water-Exposure Outcome
1	0.897 ± 0.079	4.61 ± 0.92	2.77 ± 2.22	237.5 ± 133.3	0/5 measurable at 2 h
2	0.688 ± 0.049	2.07 ± 0.39	1.90 ± 0.83	278.0 ± 144.0	3/5 measurable at 168 h
3	0.705 ± 0.061	1.60 ± 0.44	3.19 ± 2.44	593.5 ± 444.7	1/5 measurable at 168 h
4	0.891 ± 0.049	5.73 ± 1.23	4.17 ± 0.95	547.8 ± 113.9	4/5 measurable up to 72 h 0/5 measurable at 168 h
6	0.889 ± 0.090	3.53 ± 1.83	4.64 ± 2.07	626.7 ± 284.7	4/5 measurable at 168 h
7	0.959 ± 0.076	4.62 ± 1.72	3.38 ± 1.29	489.0 ± 187.5	0/4 measurable at 2 h

Values of apparent density and mechanical properties are expressed as mean ± standard deviation. Water-exposure outcome indicates the number of specimens that retained sufficient integrity for quantitative measurement at the specified exposure time.

## Data Availability

The original contributions presented in this study are included in the article/Supplementary Materials. Further inquiries can be directed to the corresponding author.

## Supplementary Materials

The following supporting information can be downloaded at: https://www.mdpi.com/article/10.3390/ma19204277/s1, Figure S1: Representative macroscopic images of the consolidated mycelium-based composite plates for the investigated formulations prior to specimen cutting. The images show the upper surface (facing the covering foil during pressing) and cross-sectional view through the plate thickness; Figure S2: Representative mycelium-composite specimen after three-point bending: (a) upper-surface view and (b) lower-surface view after testing; Data S1: Excel workbook containing specimen-level data for apparent density, compression, three-point bending, and water-immersion measurements. The water-immersion dataset includes specimen identifiers, scheduled observation times, mass and dimensional measurements, calculated water absorption and dimensional changes, and integrity-status records permitting verification of the retention-of-measurable-integrity data presented in Figure 4e.
