# Supplementary material for: Formulation-Dependent Physical, Mechanical, and Water-Exposure Behavior of Hemp-Based Mycelium Composites with Polymeric Additives
Source: Materials (Basel). 2026 Oct 10;19(20):4277. doi: 10.3390/ma19204277 (PMC13707017; doi:10.3390/ma19204277)
Supplement: Supplementary file 1 [file materials-19-04277-s001.zip › materials-4613443-supplementary.pdf]

Supplementary Material

Formulation-Dependent Physical, Mechanical, and Water-Exposure Behavior of Hemp-Based Mycelium Composites with Polymeric Additives

Ewa Borucinska-Parfieniuk [1], Małgorzata Kowalska [2], Madhavi H. Dalsaniya [1], Adrian Dubicki [1], Magdalena Pantol [1], Wojciech Grodzki [1], Małgorzata Krasowska [2], Jolanta Piekut [2], Eugene Yurchenko [2], Ewa Zapora [2] and Izabela B. Zglobicka [1,*]

[1] Faculty of Mechanical Engineering, Bialystok University of Technology, Wiejska 45B, 15-351 Bialystok, Poland; ewa.borucinska-parfieniuk@sd.pb.edu.pl (E.B.-P.); madhavi.dalsaniya@pb.edu.pl (M.H.D.); a.dubicki@pb.edu.pl (A.D.); magdalena.pantol@sd.pb.edu.pl (M.P.); w.grodzki@pb.edu.pl (W.G.)

[2] Faculty of Civil Engineering and Environmental Sciences, Bialystok University of Technology, Wiejska 45E, 15-351 Bialystok, Poland; malgorzata.kowalska@pb.edu.pl (M.K.); m.krasowska@pb.edu.pl (M.K.); j.piekut@pb.edu.pl (J.P.); yauheni.yurchanka@pb.edu.pl (E.Y.); e.zapora@pb.edu.pl (E.Z.)

* Correspondence: i.zglobicka@pb.edu.pl

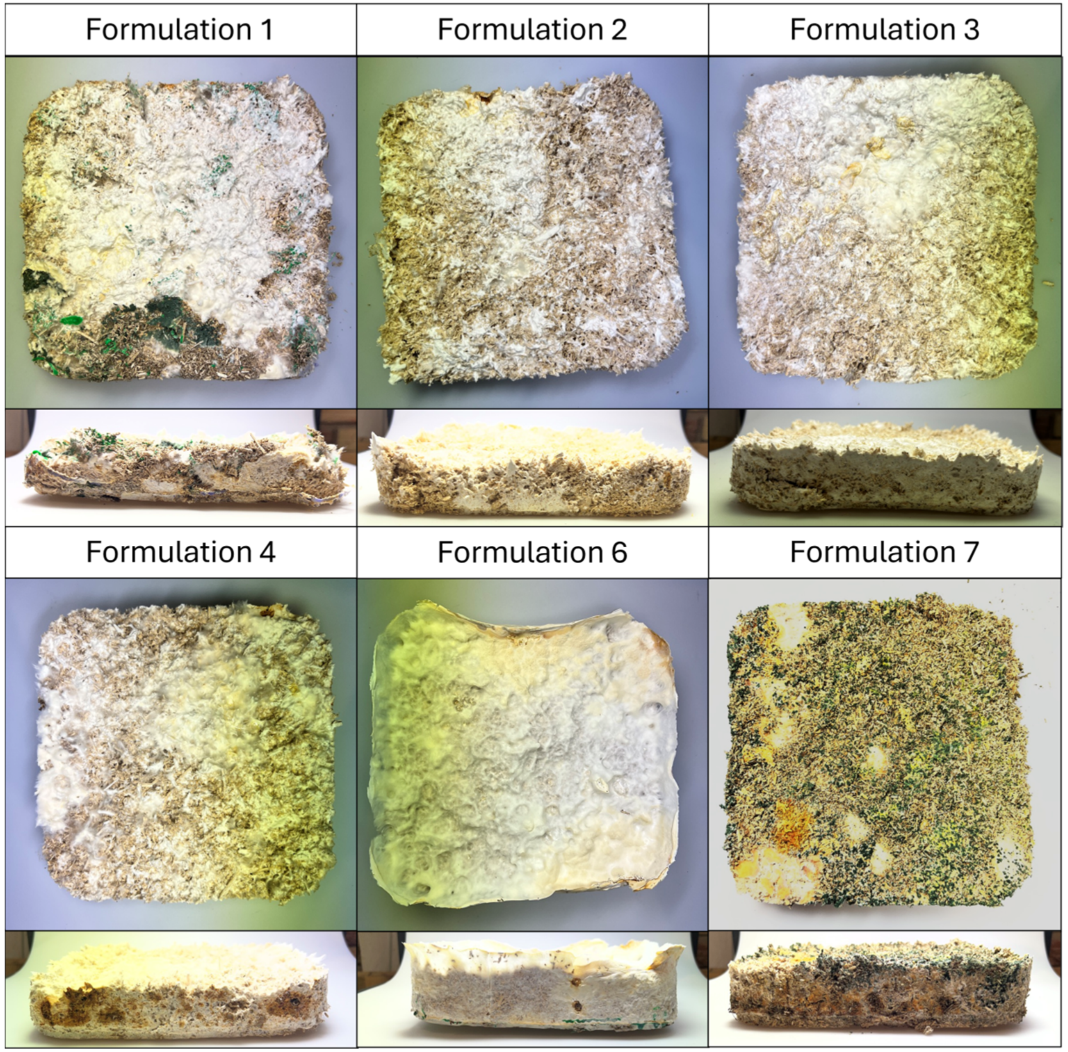

Figure S1: Representative macroscopic images of the consolidated mycelium-based composite plates for the investigated formulations prior to specimen cutting. The images show the upper surface (facing the covering foil during pressing) and cross-sectional view through the plate thickness

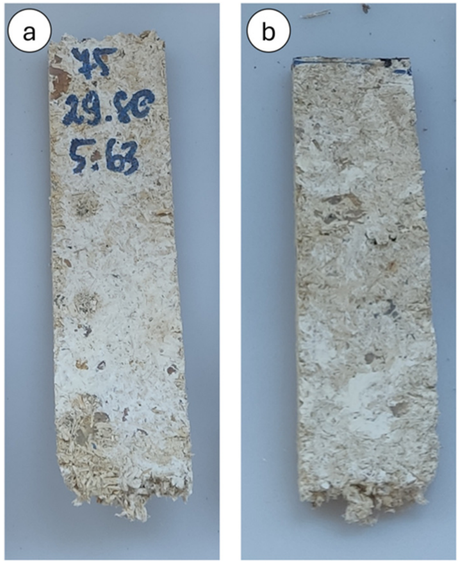

Figure S2. Representative mycelium-composite specimen after three-point bending: (a) upper-surface view and (b) lower-surface view after testing
